# Adherence to walking exercise prescription during pulmonary rehabilitation in COPD with a commercial activity monitor: a feasibility trial

**DOI:** 10.1186/s12890-021-01406-9

**Published:** 2021-01-18

**Authors:** Sarah Ward, Mark Orme, Jakub Zatloukal, Sally Singh

**Affiliations:** 1 CERS, University Hospitals of Leicester NHS Trust, Leicester, UK; 2 University of Leicester, Leicester, UK; 3 Pulmonary Rehabilitation Department, Glenfield Hospital, Groby Road, Leicester, LE3 9QP UK

**Keywords:** Physical activity, Adherence, Exercise, Rehabilitation, COPD, Activity monitor

## Abstract

**Background:**

Regular exercise is important in the management of COPD. Pulmonary rehabilitation (PR) facilitates a more physically active lifestyle through exercise participation, ideally without compromising non-exercise physical activity (PA). During PR patients are advised to perform exercise defined by duration and intensity. The extent to which PR attendees participate in unsupervised exercise bouts and their adherence to the exercise prescription provided during PR is unclear. Commercially available devices have the potential to support patients to exercise at their individually prescribed intensity. Study aims were to (1) assess how adherent patients are to their prescribed walking intensity; (2) examine the pattern of overall PA and walking exercise during the course of PR; (3) determine the feasibility of prescribing exercise to PR attendees using an activity monitor; and (4) explore the relationship between exercise and non-exercise PA with routine PR outcome measures.

**Methods:**

19 patients wore an activity monitor during routine walking tests and 6 weeks of PR, recording in a diary when they exercised. Exercise intensity (cadence) was prescribed from the Endurance Shuttle Walk Test. Patients completed questionnaires, walking tests and a lower limb strength test before and after PR. Repeated ANOVA compared changes in outcomes between weeks 1–6.

**Results:**

Patients wore the monitor every day during PR (median 42 days). Exercise steps increased by 56% (Δ332 [95% CI 54–611] steps/day, p = 0.009) between weeks 1 and 6, with no significant change in non-exercise steps (Δ79 [95% CI − 22 to − 179] steps/day, p = 0.13). Patients reported exercising on 70% of days. Adherence to prescribed cadence was achieved 55% of time spent exercising, and did not change across the 6 weeks (p = 0.907). Change in total daily steps was associated with improved dyspnea (p = 0.027), Chronic Respiratory Questionnaire (CRQ) Dyspnea domain (p = 0.019), CRQ Emotional Functioning domain (p = 0.001) and CRQ Mastery domain scores (p = 0.001) but not with exercise capacity or lower limb muscle strength.

**Conclusions:**

Improvements in exercise participation, not at the expense of non-exercise PA, throughout a PR course was observed in attendees provided with a commercially available activity monitor. Wearable technology may be able to support effective remote walking exercise prescription and participation during PR.

*Trial registration (retrospectively registered)*: http://www.isrctn.com/ISRCTN15892972.

## Background

Chronic obstructive pulmonary disease (COPD) was estimated to be responsible for 5% of deaths globally in 2015 [1] and is anticipated to become the fourth leading cause of premature mortality globally by 2030 [2]. COPD is associated with symptoms of dyspnea, sputum production, chronic cough, exacerbations and reduced exercise capacity; often culminating in poor health-related quality of life [3]. People living with COPD are not only less physically active compared to their healthy peers [4] but also compared to other chronic conditions [5, 6]. Lower levels of physical activity (PA) are associated with hospitalisation [5, 6] and an increased risk of premature death [7].

Pulmonary rehabilitation (PR) is an internationally advocated and well-established intervention for people living with COPD. PR is a low-cost, high-impact intervention broadly comprising exercise and education to break the spiral of deteriorating functional ability and worsening symptoms [8]. The traditional aim of PR is to increase exercise capacity but the most recent American Thoracic Society/European Respiratory Society’s statement on PR proposes that services also have a responsibility to encourage behavior change and promote healthy lifestyles. The long-held assumption that an increase in exercise capacity automatically translates into a more physically active lifestyle has been found to be inconsistent at best [9].

This disconnect between capacity and overall activity presents a challenge for PR programs; the traditional model includes individually prescribed exercise along with instruction and encouragement to adopt higher levels of PA as well. Programs tend to rely on self-reported adherence to exercise; subject to social desirability and recall biases. It may be possible to prescribe exercise intensity and evaluate adherence using wearable devices.

## Methods

### Study design

A single-arm feasibility study was conducted. Ethical approval was provided by NRRES committee North-East Newcastle and North Tyneside 1 (16/NE/0236). The trial was registered with ISRCTN, number ISRCTN15892972. All participants provided written informed consent. This study conforms to CONSORT guidelines and a checklist is included as Additional file 2.

### Aims

The aims of this study were to (1) assess how adherent patients are to their prescribed walking intensity; (2) examine the pattern of overall PA and walking exercise during the course of PR; (3) determine the feasibility of prescribing exercise to PR attendees using an activity monitor; and (4) explore the relationship between exercise and non-exercise PA with routine PR outcome measures.

### Setting

Participants were recruited from the PR Department at Glenfield Hospital, University Hospitals of Leicester NHS Trust, Leicester, United Kingdom between December 2016 and June 2018. Study measures were obtained before the first PR class (baseline), and after PR at the participants’ discharge assessment (post PR). There was no follow up after the discharge assessment. Demographic information was obtained from participants’ initial clinical assessment once study consent was given.

The primary outcome for this study was adherence to prescribed walking speed using an activity monitor to monitor and record cadence.

Secondary outcome measures were: usage of activity monitor (days worn from available days); adherence to exercise prescription (frequency of recorded exercise); change in PA during PR; association of exercise adherence and routine PR outcome measures (exercise capacity, endurance, quality of life measures, disease severity).

### Participants

Potential participants attending a PR assessment were approached by the clinical team and given a study patient information sheet. Were they interested in taking part, an appointment was made to consent and take study measures immediately before their planned first PR class.

Inclusion criteria were: adults with a diagnosis of COPD (FEV_1_/FEV < 0.7 measured by spirometry); stable condition, medically optimized; able and willing to comply with all PR and study requirements; able to appropriately place the activity monitor on their person; willing and able to give informed consent. Exclusion criteria were: any other significant disease or disorder which, in the opinion of the investigator, may either put the participants at risk because of participation in the study, or may influence the result of the study, or the participant’s ability to participate in the study; participant being unable to understand written or spoken English.

### Pulmonary rehabilitation

The PR programme at UHL follows the guidelines and standards set out in BTS guidelines for PR [10] and are described in detail in Additional file 1, but with specific reference to the walking component of the exercise session; participants used either indoor (corridor) or outdoor (garden) space adjacent to the rehabilitation gym to walk at their prescribed intensity, described in “Routine PR outcomes” section below. Participants were encouraged to maintain the same intensity but to progressively extend the duration of the walking. Intensity was monitored, and corrected where appropriate, by rehabilitation staff during the sessions. In addition to the supervised class sessions, participants are asked to perform walking exercise on the remaining days of the week, at the same intensity, and one unsupervised strength training session at home.

### Intervention

#### Calculating walking exercise prescription

Participants wore a Fitbit Zip™ (Fitbit, San Francisco, California, USA) activity monitor during their Endurance Shuttle Walking Test (ESWT) prior to them starting classes. Step count over the course of the ESWT (excluding warm up phase of the test), was measured via the activity monitor display. The participants’ exercise prescription (steps per minute; cadence) was then calculated by dividing total number of steps taken during the ESWT by the duration of the test.

The exercise prescription was discussed with the participant; emphasis being placed on using the activity monitors display during each exercise session to check adherence to their prescribed cadence. Opportunity was given for the participant to ask any questions. All procedures carried out during the consent visit were carried out by the researcher. The participant then went on to attend their first supervised exercise session where the clinical staff introduced them to the format of their exercise training, home exercise programme and supervised the first use of the activity monitor during their walking exercise, and documentation thereof.

An exercise diary was given to participants to document each walking exercise session. They were required to note the date, time, step count immediately prior to and after the exercise bout and duration of exercise bout, in minutes and seconds.

#### Progression of exercise during PR

During each of the participants’ subsequent supervised classes their activity monitor was “synced” to the activity monitor and Fitabase platforms. As per usual care, patients discussed their goal progression and home exercise with a PR therapist. Either the researcher or a PR therapist then fed back the data reflecting on adherence to wear, adherence to their exercise regime, achievement of prescribed cadence during exercise bouts, agreement of activity monitor data with diary entries and any issues the participant may be having with the activity monitor, or its use. During the first two sessions, the physiotherapist would discuss and agree a target for their exercise bouts for the coming week. Once a full week of step data was available a target for total daily steps, including those accrued during exercise bouts, were agreed with the patient.

Participants achieving exercise bout step target ≥ 5 days of the previous week; exercise bout step target increased by 5% of previous target. Participants achieving PA step target ≥ 4 days of the previous week: step count relating to non-exercise PA (total daily step count minus exercise step count) increased by 10%. The PA target then added to the exercise bout target became the participants’ new PA target for the coming week. The decision on how to increase both exercise and total step count targets was made at the beginning of the study; as a way of encouraging participants to be consistent with increases in exercise steps we felt that identifying a step target for exercise bouts and a separate one for overall daily PA would help. This was considered to be a reasonable way to achieve both the aims described above and is supported by other published material [11].

### Physical activity monitoring

#### Physical activity data capture

Participants were asked to wear the validated [12–14] Fitbit Zip™ activity monitor, on their trouser waistband, front trouser pocket or bra, according to manufacturer recommendations. Participants were requested to wear the activity monitor every day for the full duration of their PR programme, removing it for sleep and any water-based activities. Step count data were extracted in 60-s epochs from Fitabase (Fitabase, San Diego, California USA). All Fitbit device accounts were set up using study-specific email accounts. Data from the activity monitors were synced at each PR class to avoid data loss and to enable the extraction of raw data.

#### Physical activity variables

Adherence to wearing the activity monitor was considered successful if the activity monitor was worn for 5 days out of seven each week of the participant’s PR programme. Exercise sessions were identified based on whether step count data could be identified from the Fitabase export at the same time as and with the same number of steps as recorded in the participants’ exercise diary. To account for the possibility of accidental errors in self-reported timings, a ten-minute margin of error was accepted when identifying exercise bouts from the Fitabase export.

For each bout of walking exercise, the percentage of time performed at the prescribed cadence was calculated. Daily step count was extracted and subdivided into steps taken as part of recorded exercise and steps taken as non-exercise PA.

### Routine PR outcomes

Measures completed by the PR clinical team during the initial clinical assessment included disease severity using the Medical Research Council scale (MRC) [15] and breathlessness at rest rating [16]. Exercise capacity and endurance were measured using the Incremental Shuttle Walking Test (ISWT) [17] and ESWT [18], respectively. Endurance speed was set at 85% predicted V0_2_peak, determined from the ISWT. Quadriceps muscle strength was assessed using a strain gauge, measured in kilograms. Health related quality of life was measured using the Chronic Respiratory Disease Questionnaire-Self Report (CRQ-SR) [19].

### Blinding to outcome measures

It was not possible to blind the participants to the intervention as they would need to engage with using the activity monitor. Similarly, the nature of analyzing the activity monitor data, formulating feedback and goal setting with participants meant that blinding the assessor was also not possible.

### Statistical analysis

Data are presented as frequencies and percentage (%), mean (SD) or median (IQR) based on its distribution. The relationship between step count measured by activity monitor and direct observation during the baseline ESWT was analyzed using a Spearman’s correlation, Bland–Altman plot [20, 21] and mean absolute percentage error (MAPE). Weekly exercise intensity adherence was tested with repeated measures analysis of variance. Due to the small sample size non-parametric statistical tests were used for within group changes between week 1 and 6. Associations between changes in PA variables with routine PR outcomes were assessed using Pearson’s or Spearman’s correlations.

No formal sample size calculation was undertaken for this feasibility study, however consideration was given to how many potentially suitable participants might be identified by the clinical service, how many might consent to enrol and an estimated dropout rate across the time frame of the study to inform on the size of sample, which was set at 20 participants.

## Results

**Fig. 1:**
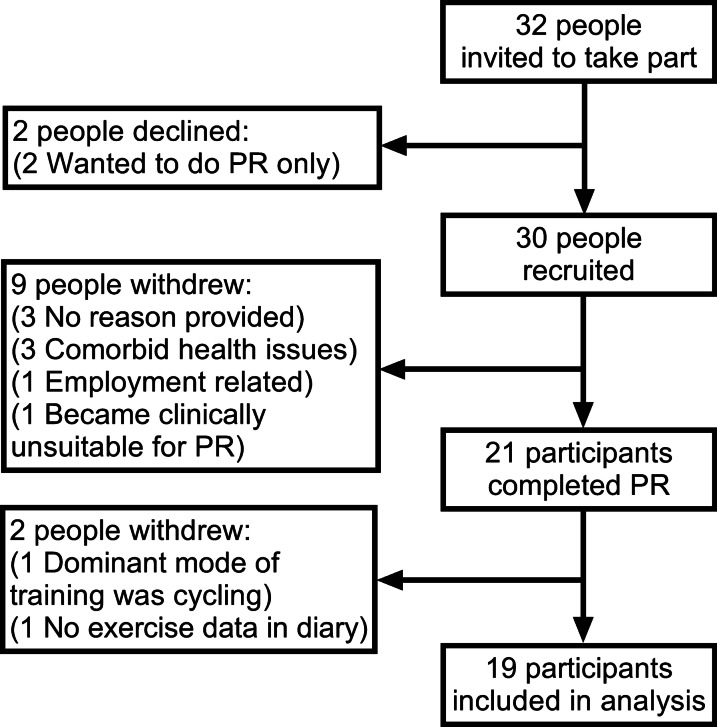
Flow of participants through the study

Thirty-two patients were interested in taking part in the study, two subsequently declined and so thirty participants consented onto the study and 21 completed a programme of PR (Fig. 1). Step count data was excluded from two participants; one participant was excluded from analysis as the dominant mode of training for this participant was cycling, not walking, rendering the ‘step counts’ invalid. The second participant was excluded as they did not record any data in their exercise diary. Baseline characteristics are provided in Table 1.

**Table 1:** Baseline characteristics

Baseline characteristics	Mean (SD)
Age, years	66.3 (7.0)
Male sex (%)	11 (58)
FEV_1_%predicted	40.8 (15.7)
*MRC grade*	
1/2/3/4/5 (%)	0/3/7/7/2 (0/16/37/37/11)
BMI	27.3 (6.1)
Oxygen use (%)	3 (16)
*Smoking status*	
Current/former/never (%)	0/18/1 (0/95/5)
ISWT (m) median (IQR)	280 (220)
ESWT (sec)	249 (113)
QMVC (kg)	19.6 (8.7)
CRQ dyspnoea	2.5 (0.8)
CRQ fatigue	3.2 (1.1)
CRQ emotion	4.3 (1.0)
CRQ mastery	4.4 (1.1)

N = 19. Data are reported as mean (SD) unless otherwise stated

*BMI* Body Mass Index, *CRQ* Chronic Respiratory Disease Questionnaire, *ESWT* endurance shuttle walk test, *FEV*_*1*_*%* forced expiratory volume in 1 s % predicted, *ISWT* incremental shuttle walking test, *MRC* Medical Research Council, *QMVC* quadriceps maximal voluntary contraction

### Feasibility of prescribing exercise intensity using activity monitors

**Fig. 2:**
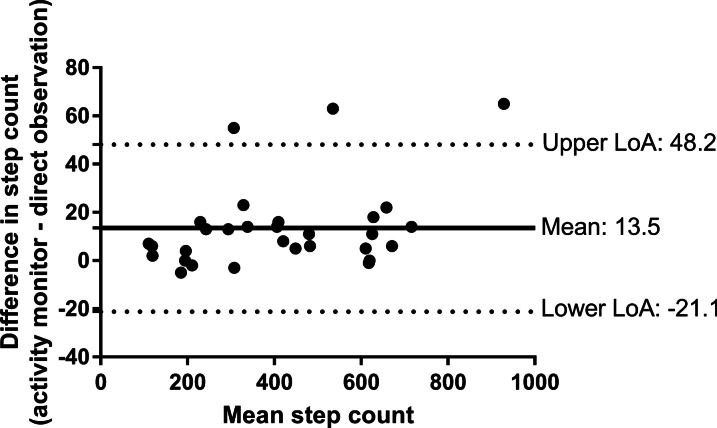
Bland–Altman plot showing agreement between step counts measured by activity monitor and direct observation during ESWT. *ESWT* endurance shuttle walk test, *LoA* limit of agreement

All participants were prescribed an exercise intensity using cadence from an activity monitor; with the average ESWT level achieved during the baseline testing was level 8 (range 2–16; equating to a walking speed of 3.79 km/h), and an mean (SD) cadence of 101 (17) steps/min (range 68–128 steps/min). Correlation between the steps measured by the activity monitor and direct observation was high (Spearman’s ρ = 0.991, p < 0.01). Overall, the activity monitors overestimated total step accumulation during a walking test by 13.5 steps (MAPE 3.3%), (Fig. 2).

### Use of the activity monitors to walk at the prescribed intensity

**Fig. 3:**
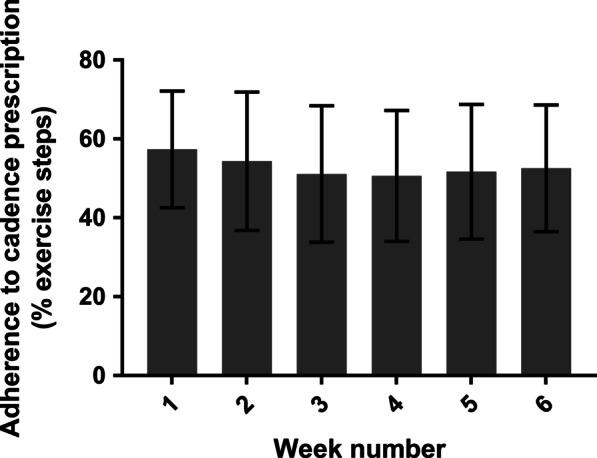
Adherence to prescribed walking exercise (cadence). Adherence calculated as the proportion of time spent in cadence equal to or greater than their exercise prescription. Data presented as mean (95% confidence interval)

The activity monitor was worn a median of 42 (0) days and patients reported exercising whilst wearing the activity monitor a median of 35 (IQR 19) days. Adherence to prescribed cadence was achieved for 53.2% (95% CI 39.9–66.6%) of time spent exercising, and was consistent across the 6 weeks (p = 0.907, Fig. 3).

### Change in PA and walking exercise during PR

**Fig. 4:**
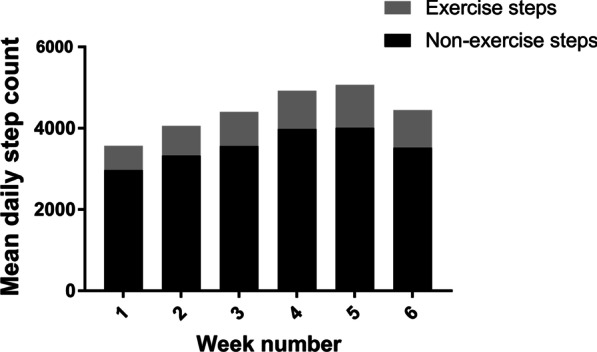
Mean daily step count and mean exercise step count change across weeks 1–6 of PR

There was an increase in total daily steps by 20% from week 1 to week 6 (week 1: 3565 [95% CI 2779–4351] vs week 6: 4447 [95% CI 3333–5561] steps/day, p = 0.036). Exercise steps increased by 56% (week 1: 595 [95% CI 397–793] vs week 6: 927 [95% CI 599–1256] steps/day, p = 0.009) between weeks 1 and 6, with no significant change in non-exercise steps (week 1: 2970 [95% CI 2251–3689] vs week 6: 3520 [95% CI 2580–4459] steps/day), p = 0.13) (Fig. 4).

### Relationship between exercise adherence and PR outcomes

Post PR assessment showed statistically significant improvements in all the routine PR measures, Additional file 3. Significant correlations between the change in total daily steps and clinical outcome measures were observed for changes in MRC scale (p = 0.027), CRQ Dyspnea (p = 0.019), CRQ Emotional Functioning (p = 0.001) and CRQ Mastery scores (p = 0.001) (Table 2). Change in daily exercise steps and clinical outcomes were significantly correlated with changes in MRC scale (p = 0.013), CRQ Dyspnea (p = 0.021), CRQ Emotional Functioning (p = 0.018) and CRQ Mastery (p = 0.003). Significant correlations between the change in daily non-exercise steps and clinical outcomes was observed for changes in CRQ Dyspnea (p = 0.047), CRQ Emotional Functioning (p = 0.002) and CRQ Mastery scores (p = 0.006).

**Table 2:** Correlation between exercise adherence and PR outcomes

Change	Change in daily steps	Change in exercise steps	Change in non-exercise steps	Percentage of exercise at cadence
ISWT (m)	0.08	0.05	0.08	0.25
ESWT (s)	0.36	0.45	0.27	0.36
QMVC (kg)	0.02	0.01	0.03	0.14
MRC grade	**− 0.58**	**− 0.69**	**− **0.45	**− **0.11
CRQ dyspnoea	**0.53**	**0.53**	**0.46**	0.19
CRQ fatigue	0.23	0.25	0.18	0.17
CRQ emotional	**0.69**	**0.54**	**0.66**	**− **0.17
CRQ mastery	**0.68**	**0.65**	**0.60**	0.19

Data presented as correlation coefficients. Bold signifies *p* < 0.05. *CRQ* Chronic Respiratory Disease Questionnaire, *ESWT* endurance shuttle walk test, *ISWT* incremental shuttle walkinging test, *MRC* Medical Research Council, *QMVC* quadriceps maximal voluntary contraction

## Discussion

### Main findings

It is possible to individually prescribe exercise using cadence calculated from a commercially available activity monitor and patients living with COPD will wear an activity monitor throughout a 6-week PR course, including using it during their exercise in an attempt to walk at their individually prescribed pace. Increases in their walking exercise participation without subsequent reductions in non-exercise PA were observed, but approximately 50% of exercise steps were not taken at the prescribed intensity. An increase in PA during PR was associated with improvements in self-reported measures of symptom burden. Efforts to improve the adherence to prescribed walking exercise during PR are needed.

### Adherence to prescribed walking intensity

It was possible to prescribe personalized exercise intensity using an activity monitor when worn during a constant work-rate field walking test (ESWT).The present study identified that although participants wore the activity monitor to try to monitor intensity of exercise, approximately half the walking exercise steps recorded by participants were completed at an intensity below their PR exercise prescription. This finding is support by Hunter, Singh and Morgan [22] who also found a 50% adherence when using a subjective measure of exercise intensity combined with an activity monitor (with no patient feedback) during PR. There have been other approaches to provide additional support to patients to facilitate optimal home-based exercise. For example, Liu et al. [23] found that people living with COPD who listened to music of an appropriate tempo to pace their exercise showed an increase in exercise capacity and maintained their exercise frequency across a nine-month self-management period.

In routine clinical practice in the UK, controlling a patient’s walking intensity during a PR programme typically relies on self-documented exercise diaries and perceived effort/breathlessness. The combination of duration and end of exercise perceived effort, or breathlessness, will inform the clinician of whether the intensity of exercise is in line with that prescribed. In the present study the initial pacing, correction and supervision of cadence was carried out by experienced members of the clinical team with many accumulated years of experience communicating this type of exercise to PR patients. Despite this, the level of adherence to an exercise prescription also did not improve during the six-week programme, suggesting that the opportunity to correct exercise adherence at the supervised exercise sessions was either missed, or did not help patients translate this to their unsupervised home sessions. Strategies to facilitate adherence to home-based walking exercise are needed.

As wearable technologies develop there may be more options to allow patients to make real-time adjustments during their exercise session to maintain the appropriate intensity for longer. The high adherence to wearing the activity monitor in this study supports the potential for wearable technologies to play a role in the context of PR but the practicalities of integrating wearable technologies into NHS clinical services remains a challenge.

The notable drop-off in PA between week 5 and week 6 observed in the present study may suggest that PR attendees begin to ‘ease off’ their exercise routine towards the end of the programme, which may help explain previous observations that patients revert to baseline behaviours [24, 25]. This may be because PR attendees struggle to understand, or buy into, the necessity of long-term adherence to regular exercise or it may be that PR does not activate patients sufficiently by the end of the programme. Changing complex behavior patterns is likely to require a multifaceted approach, as described by Spruit et al. [26] in their concise clinical review of PR and PA in patients with COPD. PR gives patients the capacity to do more and potentially the self-efficacy to be encouraged to try more, yet this does not automatically translate into an increase in activity for these patients [26].

### The pattern of exercise and non-exercise walking during the course of PR

This present study was able to show a 20% (~ 900 steps per day) increase in PA, primarily driven by an increased step count amassed through exercise, and exceeding the minimal important difference for PA [27]. An increase in PA is a desirable outcome for PR [28] but there has been considerable heterogeneity in whether PR leads to an increase in physical activity [28, 29]. A systematic review and meta-analysis looking at interventions effective at changing a person’s PA identified PA counselling as an effective strategy [29] but it is unclear what behavior change strategies will be most effective for PR attendees to improve their exercise adherence and how best to maintain higher levels of physical activity after PR. It might be noted that the increase in exercise step count observed in this study in absolute terms (average 330 steps) seems small. However, when considering the average cadence for the group was 101steps/min and the average walking time at baseline was 4mins, an average increase in walking time of slightly more than 3minutes (75% increase) might seem very significant to individuals living with restricted activity.

### Relationship with routine outcome measures

Changes in PA were correlated with improvements in breathlessness and other quality of life measures, but not improvements in exercise capacity. It may be that symptoms, rather than improvements in exercise capacity, drive the change in PA behavior. These results contradict previous work which suggests that baseline exercise capacity predicts the PA response to PR [30]. Key methodological differences may in part explain this difference as the present study measured PA during PR rather than a before-and-after design. It is unclear when is the most appropriate time to evaluate the complex behavior that is PA in the context of a complex intervention that is PR, and differences between protocols may contribute to heterogeneous findings in the literature. Data on adherence to exercise during PR is sparse and the present study shows that the dose of exercise taken by patients is lower than the prescription, highlighting a potential target for interventions to enhance the short- and long-term benefits of PR.

### Limitations

There are limitations to this study which must be accounted for when interpreting the results. There is no measurement of participants’ usual step counts before they started their course of PR preventing a pre-post PR comparison of physical activity. However, since the study was specifically looking at exercise during the course of a rehabilitation programme, the absence of pre-PR baseline step does not impact the overall interpretation of the results obtained. The absence of a control group prevents causal inferences. Whilst this has no bearing on the findings on patient adherence to wearing an activity monitor or their adherence to walking exercise prescription during PR, it does mean the present study is unable to rule out the Hawthorne effect. That said if measurement reactivity has occurred in present study, the finding of 50% adherence to exercise intensity would be an overestimation of adherence. The absence of qualitative methods prevented further understanding of patient perceptions of home-based exercise and explore why they were unable to consistently adhere to the prescribed walking intensity. It is known that there are specific barriers and enablers to taking on and completing a course of PR, as well as undertaking physical activity in general [31], and it might be reasonable to assume these are similar. This study is looking at exercise participation, rather than general physical activity however there is likely to be some overlap; such as with weather, daylight hours, perceived safety etc. There may be other barriers more specific to participating in an exercise session; the terrain in the area the individual is able to access for exercise such as uneven pavements, urban areas with lots of road crossings; hills and slopes that form part of the individuals’ local exercise area, for example. Finally, the present study only recruited people living with COPD and so may not be generalizable to other conditions undergoing rehabilitation.

## Conclusion

It is possible to prescribe walking exercise intensity using cadence calculated using a commercial activity monitor worn during a routinely measured field-based walking test and patients attending PR wore the activity monitor during their exercise in a bid to walk at their prescribed pace. Exercise participation increased during the 6-week PR programme, without compromising non-exercise PA and was associated with improvements in measures of symptom burden. Wearable technology may be able to support effective remote walking exercise prescription and participation during PR. Patients’ adherence to a prescribed exercise intensity may be lower than anticipated by PR professionals but it was positive to see that this was not at the expense of non-exercise activities.

## Supplementary information

**Additional file 1.** Description of UHL Pulmonary Rehabilitation Programme.

**Additional file 2.** Completed CONSORT checklist for this study.

**Additional file 3.** Table showing the change in croutine clinical outcomes following PR.

## Data Availability

The datasets used and analysed in the current study are available from the corresponding author on reasonable request.

## Abbreviations

COPD: Chronic obstructive pulmonary disease
CRQ-SR: Chronic respiratory questionnaire-self-reported
ESWT: Endurance shuttle walking test
FEV_1_/FVC: Ratio of forced expiratory volume in 1 s and functional vital capacity
IQR: Interquartile range
ISWT: Incremental shuttle walking test
MAPE: Mean absolute percentage error
MRC: Medical Research Council
PA: Physical activity
PR: Pulmonary rehabilitation
QMVC: Quadriceps maximal voluntary contraction
SD: Standard deviation
UHL: University Hospitals of Leicester
