# Supplementary material for: Adherence to walking exercise prescription during pulmonary rehabilitation in COPD with a commercial activity monitor: a feasibility trial
Source: BMC Pulm Med. 2021 Jan 18;21:30. doi: 10.1186/s12890-021-01406-9 (PMC7812648; doi:10.1186/s12890-021-01406-9)
Supplement: Supplementary file 1 — Additional file 1. Description of UHL Pulmonary Rehabilitation Programme. [file 12890_2021_1406_MOESM1_ESM.docx]

The pulmonary rehabilitation programme at UHL is twelve sessions in length; two visits weekly for approximately six weeks. The programme is a rolling one; patients starting each week once assessed. As such, should a patient be unable to attend a scheduled session due to illness or other commitments, they could remain enrolled until they had attended twelve sessions.

Each supervised session consisted of one hour of exercise and up to one hour of multidisciplinary education. Exercise included cardiovascular training; walking at endurance speed set at assessment and static cycling at a resistance level to achieve five minutes of continuous work; functional resistance training of upper and lower limbs with dumbbells. The weight of the dumbbells was set such that participants can achieve three sets of ten repetitions or each exercise. Education sessions were delivered or facilitated by the multidisciplinary team and topics included disease education, medicines management and inhaler use, symptom management, energy conservation, nutrition, airway clearance, anxiety management, exacerbation awareness and avoidance and community opportunities for exercise, activity and support groups. Written information relating to the education discussions are also provided to participants, either in the form of published information from appropriate sources such as the British Lung Foundation, or produced by the PR department itself.

On completion of the PR course participants attended an individual discharge assessment where measures of exercise capacity, endurance, muscle strength and quality of life were repeated. At this point referrals to community-based services providing maintenance of exercise regimes were made at participants’ request. Regardless of any onward referrals, all participants were given a written plan of on-going exercise from the researcher carrying out the discharge assessment.
