# Supplementary material for: Adherence to walking exercise prescription during pulmonary rehabilitation in COPD with a commercial activity monitor: a feasibility trial
Source: BMC Pulm Med. 2021 Jan 18;21:30. doi: 10.1186/s12890-021-01406-9 (PMC7812648; doi:10.1186/s12890-021-01406-9)
Supplement: Supplementary file 3 — Additional file 3. Table showing the change in croutine clinical outcomes following PR. [file 12890_2021_1406_MOESM3_ESM.docx]

Table to show the change in routine outcome measures after PR

| Routine Clinical Outcome | Baseline | Mean change | p-value |
| --- | --- | --- | --- |
| ISWT m ***** | 280 (220) | 50 (58) | 0.001 |
| ESWT sec | 249 (112.62) | 300 (360.35) | 0.003 |
| QMVC kg | 19.56 (8.73) | 4.46 (6.07) | 0.027 |
| MRC ***** | 3 (1) | 0 (1) | 0.024 |
| CRQ Dyspnoea | 2.48 (0.77) | 1.2 (0.99) | <0.001 |
| CRQ Fatigue | 3.22 (1.11) | 1.27 (1.40) | 0.001 |
| CRQ Emotional Functioning | 4.35 (0.96) | 0.92 (0.82) | <0.001 |
| CRQ Mastery | 4.39 (1.04) | 0.99 (1.16) | <0.001 |

All measures are reported as Mean (SD) unless otherwise stated. * Median and IQR
